# Naturally Occurring Lipid A Mutants in *Neisseria meningitidis* from Patients with Invasive Meningococcal Disease Are Associated with Reduced Coagulopathy

**DOI:** 10.1371/journal.ppat.1000396

**Published:** 2009-04-24

**Authors:** Floris Fransen, Sebastiaan G. B. Heckenberg, Hendrik Jan Hamstra, Moniek Feller, Claire J. P. Boog, Jos P. M. van Putten, Diederik van de Beek, Arie van der Ende, Peter van der Ley

**Affiliations:** 1 Laboratory of Vaccine Research, Netherlands Vaccine Institute, Bilthoven, The Netherlands; 2 Department of Immunology and Infectious Diseases, Utrecht University, Utrecht, The Netherlands; 3 Department of Neurology, Center for Infection and Immunity Amsterdam (CINIMA), Academic Medical Center, Amsterdam, The Netherlands; 4 Department of Medical Microbiology, Academic Medical Center, Amsterdam, The Netherlands; 5 Netherlands Reference Laboratory for Bacterial Meningitis, Academic Medical Center, Amsterdam, The Netherlands; University of Illinois, United States of America

## Abstract

*Neisseria meningitidis* is a major cause of bacterial meningitis and sepsis worldwide. Lipopolysaccharide (LPS), a major component of the Gram-negative bacterial outer membrane, is sensed by mammalian cells through Toll-like receptor 4 (TLR4), resulting in activation of proinflammatory cytokine pathways. TLR4 recognizes the lipid A moiety of the LPS molecule, and the chemical composition of the lipid A determines how well it is recognized by TLR4. *N. meningitidis* has been reported to produce lipid A with six acyl chains, the optimal number for TLR4 recognition. Indeed, meningococcal sepsis is generally seen as the prototypical endotoxin-mediated disease. In the present study, we screened meningococcal disease isolates from 464 patients for their ability to induce cytokine production *in vitro*. We found that around 9% of them were dramatically less potent than wild-type strains. Analysis of the lipid A of several of the low-activity strains by mass spectrometry revealed they were penta-acylated, suggesting a mutation in the *lpxL1* or *lpxL2* genes required for addition of secondary acyl chains. Sequencing of these genes showed that all the low activity strains had mutations that inactivated the *lpxL1* gene. In order to see whether *lpxL1* mutants might give a different clinical picture, we investigated the clinical correlate of these mutations in a prospective nationwide observational cohort study of adults with meningococcal meningitis. Patients infected with an *lpxL1* mutant presented significantly less frequently with rash and had higher thrombocyte counts, consistent with reduced cytokine induction and less activation of tissue-factor mediated coagulopathy. In conclusion, here we report for the first time that a surprisingly large fraction of meningococcal clinical isolates have LPS with underacylated lipid A due to mutations in the *lpxL1* gene. The resulting low-activity LPS may have an important role in virulence by aiding the bacteria to evade the innate immune system. Our results provide the first example of a specific mutation in *N. meningitidis* that can be correlated with the clinical course of meningococcal disease.

## Author Summary

*Neisseria meningitidis* is a Gram-negative bacterium that can cause the life-threatening diseases meningitis and sepsis. Most of the symptoms seen in these diseases are the result of excessive stimulation of the immune system by lipopolysaccharide (LPS), a major component of the outer membrane of Gram-negative bacteria. The structure of the membrane-anchoring part of LPS, named lipid A, determines how well it is recognized by TLR4, the receptor which triggers this response. *N. meningitidis* has been shown previously to make a highly active form of LPS with six fatty acyl chains in its lipid A moiety. We now show that a surprisingly large fraction of meningococcal patient isolates have a mutation in one of their lipid A biosynthesis genes, which makes their LPS underacylated and which strongly decreases their capability to activate the immune system. Presumably, selection of these mutants is promoted because the reduced activation aids the bacteria to escape elimination within the human host. We further demonstrate that meningitis patients infected with these LPS mutants have less severe symptoms than patients infected with wild-type strains. This is the first instance where a specific bacterial mutation can be correlated with the clinical course of meningococcal disease.

## Introduction

*Neisseria meningitidis* is a major cause of bacterial meningitis and sepsis worldwide [1]. While it is a frequent commensal of the human upper respiratory tract, in some individuals the bacterium spreads to the bloodstream causing meningitis and/or sepsis, serious conditions with high morbidity and mortality. As in all Gram-negative bacteria, lipopolysaccharide (LPS) is a major component of the outer membrane of *N. meningitidis*. It is now well established that LPS is sensed by mammalian cells through Toll-like receptor 4 (TLR4), in combination with coreceptors MD-2 and CD14 [2]. Activation of this complex leads to recruitment of the adapters MyD88, Mal, TRIF, and TRAM to the cytoplasmic domain of TLR4 [3]. These adapters initiate signal transduction pathways that lead to induction of innate immunity. These pathways are classified in a so called “MyD88-dependent” pathway involving MyD88 and Mal, and a “MyD88-independent” pathway involving TRIF and TRAM. Hallmarks of MyD88-dependent and MyD88-independent signaling are induction of pro-inflammatory cytokines and type I IFN respectively. While the response to LPS can be beneficial to the host by containing a beginning infection, it can also be detrimental when excessive stimulation occurs through growth of large numbers of bacteria in the bloodstream as happens during sepsis [2],[4],[5].

TLR4 recognizes the lipid A moiety of the LPS molecule [2]. The chemical composition of the lipid A determines how well it is recognized by TLR4 and consequently it determines the biological activity of the LPS. *N. meningitidis* has been reported to produce lipid A with six acyl chains, the optimal number for TLR4 recognition [6]. Indeed purified LPS of this bacterium is highly active and plasma concentrations of LPS in patients with meningococcal disease correlate strongly with mortality risk [7]. LPS is also important in the activation of the coagulation system through upregulation of tissue factor. Excessive activation of the coagulation system can lead to disseminated intravascular coagulation (DIC), the most feared complication of invasive meningococcal disease [1]. DIC is clinically characterized by hypotension, petechial rash, and depletion of thrombocytes and coagulation factors.

Uniquely among Gram-negative bacteria, *N. meningitidis* can grow without LPS, as was shown by us when we constructed a mutant with an inactivated *lpxA* gene, required for the first step in LPS biosynthesis [8]. In addition, we have previously shown that insertional inactivation of the *lpxL1* or *lpxL2* genes required for addition of secondary acyl chains leads to reduced biological activity of meningococcal LPS [9],[10]. The possibility that such mutations might also occur naturally was suggested to us by a report showing that the group Y strain HF13 was defective in signaling through the MyD88-independent pathway and TLR4 [11].

Here we report that strain HF13 has penta-acylated lipid A due to a mutation in its *lpxL1* gene. Screening of a selection of clinical isolates revealed *lpxL1* mutations in approximately 13% of meningococcal disease isolates of all major serogroups and clonal complexes. Several different kinds of mutations were found. We also found evidence for on-and-off switching of *lpxL1 in vivo* in humans. Importantly, patients with meningococcal meningitis that were infected with an *lpxL1* mutant strain had less severe systemic inflammation and reduced coagulopathy.

## Results

### Strain HF13 is a natural *lpxL1* mutant

**Figure 1:**
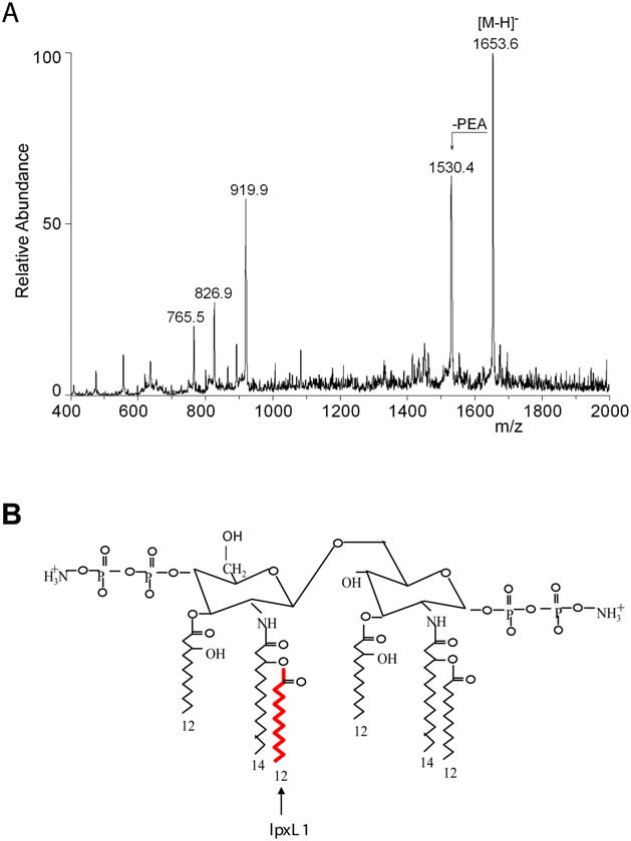
Strain HF13 is an *lpxL1* mutant. (A) Mass spectrum of HF13 lipid A. The highest peak (1653.6) corresponds to penta-acylated lipid A with two phosphate groups and one phosphoethanolamine (PEA); the second peak (1530.4) corresponds to penta-acytlated lipid A with two phosphate groups without PEA. (B) Depiction of *N. meningitidis* wild-type lipid A. The acyl chain that is added by LpxL1 is indicated.

**Figure 2:**
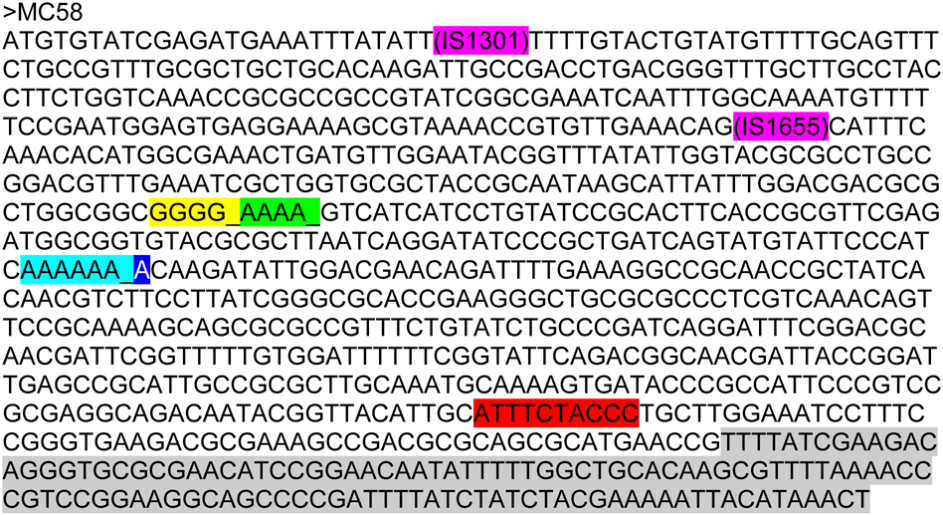
Presentation of all *lpxL1* mutations. The *lpxL1* gene sequence of strain MC58 is shown including the different types of mutations and their positions in the gene found among isolates from patients. Each type of mutation is indicated with a different color. An ‘_’ indicates a nucleotide that is deleted in the mutant strain, pink indicates an insertion element (type I and II mutations), yellow indicates a deletion of a guanine in a stretch of five guanines (type III mutation), green indicates a deletion of an adenosine in a stretch of five adenosines (type IV mutation), light blue indicates a deletion of an adenosine in a stretch of seven adenosines (type V mutation), dark blue indicates an insertion of an adenosine in a stretch of seven adenosines (type V mutation), and the sequences that are highlighted in red or gray are deleted in the mutant strain (type VI and VII mutations respectively).

Mogensen et al. demonstrated that the serogroup Y strain HF13 is defective in TLR4 activation and initiation of MyD88-independent signaling [11]. Reduced biological activity of meningococcal LPS is associated with altered lipid A structure [9],[10]. Therefore, the lipid A structure of strain HF13 was assessed by mass spectrometry (Figure 1A). The spectrum shows major peaks that correspond with lipid A with only five acyl chains. One of the two secondary C_12_ acyl chains is absent, but the spectrum is not conclusive on which one, since the C_12_ acyl chains have the same mass. This result implies that in strain HF13 either *lpxL1* or *lpxL2* is inactive, as we previously found that the addition of the secondary C_12_ acyl chains to lipid A requires active *lpxL1* and *lpxL2* [9]. Sequence analyses of both genes showed a normal *lpxL2* sequence, but the *lpxL1* sequence contained one adenosine deletion in a poly adenosine tract, leading to a frameshift and a premature stop of the translated protein (Figure 2, Table 1).

**Table 1:** List of all *lpxL1* mutant strains.

Strain no.	Isolated from	serogroup	ST	Clonal complex	Type of mutation in *lpxL1* a
*lpxL1 mutants among 56 serogroup Y isolates*					
HF13	Nd^b^	Y	nd	nd	V
2011169	blood	Y	23	23	V
2040760	blood	Y	23	23	V
970455	blood	Y	nd	nd	V
2050913	joint puncture	Y	2786	23	V
971523	CSF	Y	nd	nd	V
971886	CSF	Y	nd	nd	VI
982195	blood	Y	nd	nd	VI
2040608	CSF	Y	nd	nd	I
*lpxL1 mutants among 114 isolates representing major serogroups and clonal complexes*					
2000569	blood	X	750	750	V
2011833	blood	C	3553	269	V
2041268	blood	B	4926	35	V
2050093	blood	B	461	461	V
2030162	blood	C	337	41/44	V
2051372	blood	B	461	461	V
2010151	blood	C	461	461	IV
2021270	CSF	C	461	461	III
2041396	CSF	B	4930	18	III
2050806	CSF	B	213	213	III
2071416	blood	Y	23	23	VI
2020799^c^	CSF	B	35	35	conserved amino acid change
2050392	CSF	B	213	213	VII
*lpxL1 mutants among multiple isolates from a single patient*					
**Patient 94176**					
941761 I	CSF	C	nd	nd	conserved amino acid change
941761 III	Troat swab	C	nd	nd	wild-type
**Patient 9707010**					
970710 I	CSF	C	nd	nd	wild-type
970710 III	nose swab	C	nd	nd	IV
**Patient 971859**					
971859 I	CSF	C	nd	nd	IV
971859 III	Throat swab	C	nd	nd	wild-type
*lpxL1 mutants among isolates from 254 patients in the prospective cohort study*					
2012202	CSF	B	41	41/44	V
2020434	CSF	C	11	11	V
991174	CSF	C	11	11	V
990576	CSF	B	571	41/44	V
991382	CSF	B	191	41/44	V
2011833	CSF	C	3553	269	V
991344	CSF	B	42	41/44	III
2000607	CSF	B	40	41/44	III
2000311	CSF	B	461	461	III
991093	CSF	B	5451	32	III
2020622	CSF	B	5458	41/44	IV
990344	CSF	B	5449	41/44	IV
2010640	CSF	B	1474	41/44	conserved amino acid change
2011334	CSF	C	11	11	II
2011764	CSF	B	303	41/44	conserved amino acid change
992008	CSF	B	146	41/44	Not detected

a Type of mutations found in *lpxL1*. Colors in parentheses correspond to colors shown in Figure 2. Type I mutation: insertion of IS1301 (pink), type II mutation: insertion of IS1655 (pink), type III mutation: deletion of a guanine in a stretch of five guanines (yellow), type IV mutation: deletion of an adenosine in a stretch of five adenosines (green), type V mutation: deletion or insertion of an adenosine in a stretch of seven adenosines (light and dark blue), type VI mutation: deletion of ten nucleotides (red), type VII mutation: deletion of C-terminal part of the *lpxL1* gene (gray). For all strains with conserved amino acid changes, the inactivation of *lpxL1* has been confirmed with analysis of the lipid A by mass spectrometry.

b Nd: not determined.

c Strain 2020799 was part of both the panel of 114 isolates representing all major serogroups and clonal complexes and the panel of 254 isolates from patients in the prospective cohort study.

The inactivated *lpxL1* gene in strain HF13 results in a penta-acylated lipid A lacking the secondary acyl chain at the 2′-position in lipid A, while *N. meningitidis* typically has a hexa-acylated lipid A (Figure 1B). These results provide an explanation for the inability of strain HF13 to activate TLR4 and to initiate MyD88-independent signaling.

### Mutations in *lpxL1* are present in several serogroups and clonal complexes

To evaluate the distribution of *lpxL1* mutations among meningococcal isolates from patients, we initially screened a panel of 56 serogroup Y meningococcal isolates for their capacity to induce the MyD88-independent cytokine IP-10 in the mouse macrophage cell line J774A.1 (Figure S1). As controls, strain H44/76 and HF13 were included. Of 56 serogroup Y isolates, eight strains induced like HF13 little or no IP-10. Sequence analyses of *lpxL1* of these isolates revealed that they all had mutations in *lpxL1*, resulting in an inactive gene. Five strains had one adenosine deletion in a poly A tract just like strain HF13 (type V mutation, Figure 2, Table 1), two strains had a deletion of ten nucleotides (type VI mutation), and one strain had an insertion of the insertion element IS1301 (Type I mutation).

These results prompted us to investigate the distribution of *lpxL1* mutations among meningococci of the major serogroups and clonal complexes. Previously, we have shown that at higher dilutions an *lpxL1* mutant induces less pro-inflammatory cytokines than wild-type *N. meningitidis* [9],[10]. To identify meningococcal isolates with mutations in *lpxL1*, isolates were tested on their capacity to induce IL-6 in the human monocytic cell line Mono Mac 6 (MM6). Of 114 isolates, representing all major serogroups and clonal complexes, 13 were found to induce low amounts of IL-6 (Figure S2). Sequence analyses of *lpxL1* showed that 12 isolates had a mutation in *lpxL1*, rendering the gene inactive (Figure 2, Table 1). Of these strains, 10 had an insertion or deletion in a polyadenosine or polyguanosine tract (type III, IV and V mutations); six of these had the same mutation as found in the majority of mutant serogroup Y strains. One strain had a type VI mutation, like in the two aforementioned serogroup Y strains. One strain had a deletion of the C-terminal part of the gene (type VII mutation). The remaining strain (2020799) had apparently no mutation in *lpxL1* that would lead to its inactivation. However, closer examination of its putative amino acid sequence showed that one amino acid was altered at a position conserved in all known *lpxL1* homologues. Therefore, the LpxL1 protein of this strain is probably nonfunctional. Indeed, we confirmed that strain 2020799 had penta-acylated lipid A by mass spectrometry (data not shown). As a control, also *lpxL1* of 34 strains that induced a normal level of IL-6 was sequenced. As expected, these strains had no mutations in *lpxL1* (data not shown). Together, seven unique *lpxL1* mutations were found among this panel of different serogroups and different clonal complexes, indicating that inactivation of *lpxL1* must have occurred multiple times independently. The results show that *lpxL1* mutations are not associated with serogroup or clonal complexes and occur also among the serogroup B and C strains, which are prevalent among isolates from patients with meningococcal disease in Europe.

### Screening of *lpxL1* mutations in a panel of multiple isolates per patient

Most of the identified *lpxL1* mutations were in nucleotide repeats of adenosines and guanosines, the type III, IV and V mutations (Figure 2, Table 1). These sequences are prone to cause slippage of the DNA polymerase during DNA replication, leading to reversible frameshift mutations. This slipped-strand mispairing is the most common mechanism of translational phase variation, the process of random and reversible on-and-off switching of a gene. Phase variation creates a phenotypically diverse population, allowing the bacterium to adapt to different microenvironments within the human host. To investigate whether *N. meningitidis* can switch *lpxL1* on-and-off we screened a panel of strains obtained from different anatomical locations within individual patients: isolates from the blood and/or cerebrospinal fluid (CSF) as well as from the throat and/or nose of 40 patients were used. The MM6 cell line was stimulated with these strains and IL-6 production was measured with ELISA. Three strains induced low levels of IL-6 compared to wild-type *N. meningitidis* (Figure S3). These isolates were from three different patients. Two strains were isolated from the cerebrospinal fluid and one strain was isolated from the throat. The other isolates of these patients induced normal levels of IL-6. The *lpxL1* genes of all isolates of these three patients were sequenced and found to be mutated in the isolates that induced low IL-6, but not in the isolates that induced normal IL-6 (Figure 2, Table 1). Two strains had a type IV mutation, which potentially is reversible. The third strain had a point mutation leading to substitution of a conserved amino acid. These results suggest that in the host the expression status of *lpxL1* of meningococci is subject to phase variation.

### *lpxL1* mutants induce less pro-inflammatory cytokines in a TLR4–dependent manner

**Figure 3:**
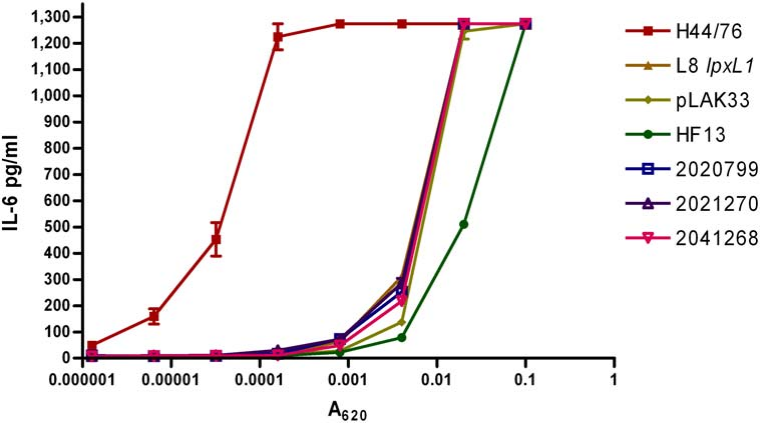
Comparison of IL-6 induction in MM-6 cells between wild-type strains and *lpxL1* mutants. MM-6 cells were stimulated for 18 h with titrations of indicated strains and IL-6 in supernatant was quantified with ELISA. H44/76 is a wild-type strain, L8 *lpxL1* is a constructed *lpxL1* mutant, pLAK33 is an LPS-deficient mutant, and all other strains are spontaneous *lpxL1* mutants. Results of one representative experiment of three independent experiments are shown. Error bars indicate S.E.M. of triplicates.

The identified *lpxL1* mutations occurred in strains of widely varying genetic background, and it is therefore conceivable that other factors besides altered LPS contribute to their reduced cytokine induction. To investigate this, titrations of four of the spontaneous *lpxL1* mutants were compared in their capacity to induce cytokines in MM6 cells with titrations of our previously constructed *lpxL1* knockout mutant and its parent strain H44/76, as well as the completely LPS-deficient strain pLAK33 (Figure 3). Clearly, the LPS-deficient strain pLAK33 is much less potent in inducing IL-6 than the wild-type strain H44/76. IL-6 induction by the constructed *lpxL1* mutant is similar to that by pLAK33 and the four *lpxL1* mutants isolated from patients.

**Figure 4:**
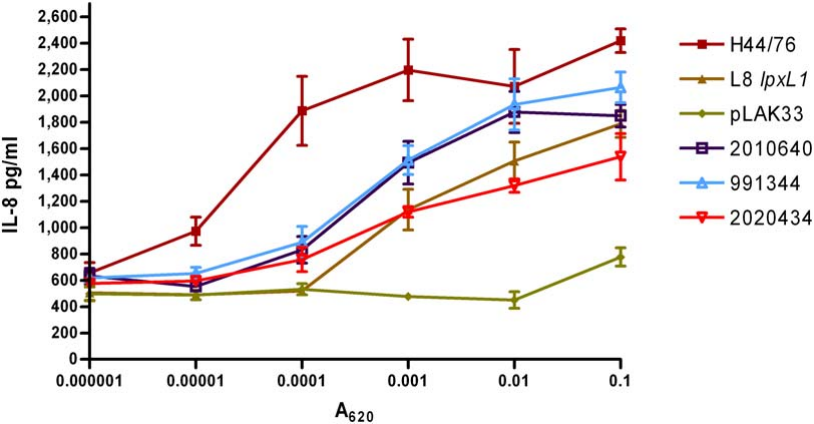
Comparison between wild-type strains and *lpxL1* mutants of IL-8 induction in HEK293 cells transfected with human TLR4. HEK293 cells transfected with human TLR4, CD14, and MD-2 were stimulated for 18 h with titrations of indicated strains and IL-8 in supernatant was quantified with ELISA. H44/76 is a wild-type strain, L8 *lpxL1* is a constructed *lpxL1* mutant, pLAK33 is an LPS-deficient mutant, and all other strains are spontaneous *lpxL1* mutants. Results of one representative experiment of three independent experiments are shown. Error bars indicate S.E.M. of triplicates.

To demonstrate that the *lpxL1* mutants induced less cytokines than wild-type strains because their LPS is less well recognized by the LPS receptor complex, titrations of a similar panel of strains was used to stimulate HEK293 cells transfected with human TLR4, MD-2, and CD14. Activation of the receptor complex was assessed by measuring IL-8 production (Figure 4). Wild-type strain H44/76 was much more efficient in TLR4 activation than the mutants. All *lpxL1* mutants, either constructed or isolated from patients, showed a similar decrease in IL-8 induction, while the LPS-deficient pLAK33 cells were even less active. Together, these results demonstrate that the *lpxL1* mutants activate human TLR4 less efficiently, and this is the sole reason for their reduced biological activity.

**Figure 5:**
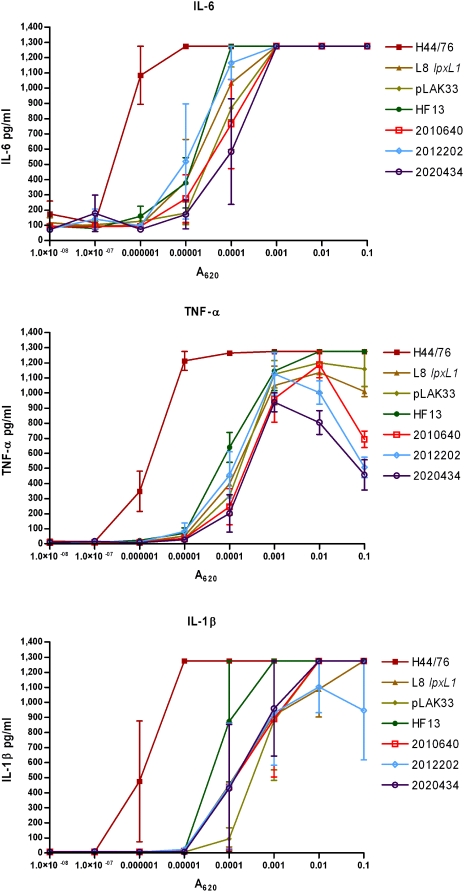
Comparison between wild-type strains and *lpxL1* mutants in pro-inflammatory cytokine induction in PBMCs. PBMCs from three different donors were stimulated with titrations of the indicated strains and IL-6, TNF-α, and IL-1β were quantified in the supernatant 18 h after stimulation. H44/76 is a wild-type strain, L8 *lpxL1* is a constructed *lpxL1* mutant, pLAK33 is an LPS-deficient mutant, and all other strains are spontaneous *lpxL1* mutants. Results of one representative experiment of two independent experiments are shown. Error bars indicate S.E.M. of triplicates.

We have shown that *lpxL1* mutants induce less cytokines in human and murine cell lines. However, these *in vitro* models do not necessarily represent the situation *in vivo* and do not take into account the genetic diversity of the human population. To mimic a systemic meningococcal infection more closely, also human peripheral blood mononuclear cells (PBMCs) of several donors were stimulated with titrations of a selection of *N. meningitidis* strains. After stimulation, concentrations of IL-6, TNF-α, and IL-1β were determined in the supernatant (Figure 5). These pro-inflammatory cytokines are known to mediate the toxic effects of LPS [2]. In all donors, wild-type strain H44/76 induced much more IL-6, TNF-α, and IL-1β than the mutants. Overall, the constructed and spontaneous *lpxL1* mutants showed a similar reduction in cytokine induction.

### Meningitis patients infected with *lpxL1* mutant meningococci have reduced inflammation and coagulopathy

We next explored whether infection with *lpxL1*-mutant meningococcal strains was associated with a particular clinical phenotype. The meningococcal isolates from 254 patients from a prospective nationwide observational cohort study of 696 adults with community-acquired bacterial meningitis in the Netherlands (period, 1998–2002) [12],[13] were analyzed for their ability to induce IL-6. Of the 254 isolates, 172 (68%) were of serogroup B, 78 (31%) of serogroup C, 3 (1%) of serogroup Y, and one (<1%) of serogroup W135. Multilocus sequence typing showed 91 unique sequence types. The most prevalent clonal complexes were cc41/44 (41%), cc11 (24%), and cc32 (16%) [13].

MM6 cells were stimulated with these strains and IL-6 induction was assessed (Figure S4). The isolates of 17 patients (7%) showed a decreased IL-6 induction and sequencing revealed mutations in *lpxL1* in all but one (Figure 2, Table 1). Twelve isolates had a type III, IV or V mutation. Three strains had a point mutation leading to substitution of an essential amino acid, and one strain had an IS1655 insertion. In one strain (992008) we were unable to identify a mutation in *lpxL1* that could lead to gene inactivation or inactive gene product. Further analyses with mass spectrometry to determine the mass of its lipid A and silver staining of a Tricine-SDS-PAGE gel to analyze the size and quantity of its LPS, demonstrated that LPS was not detectable in this strain (results not shown). The responsible mutation remains to be identified. There were no overall differences in *lpxL1* mutation frequency between serogroups (P = 0.85) and clonal complexes (P = 0.56).

**Figure 6:**
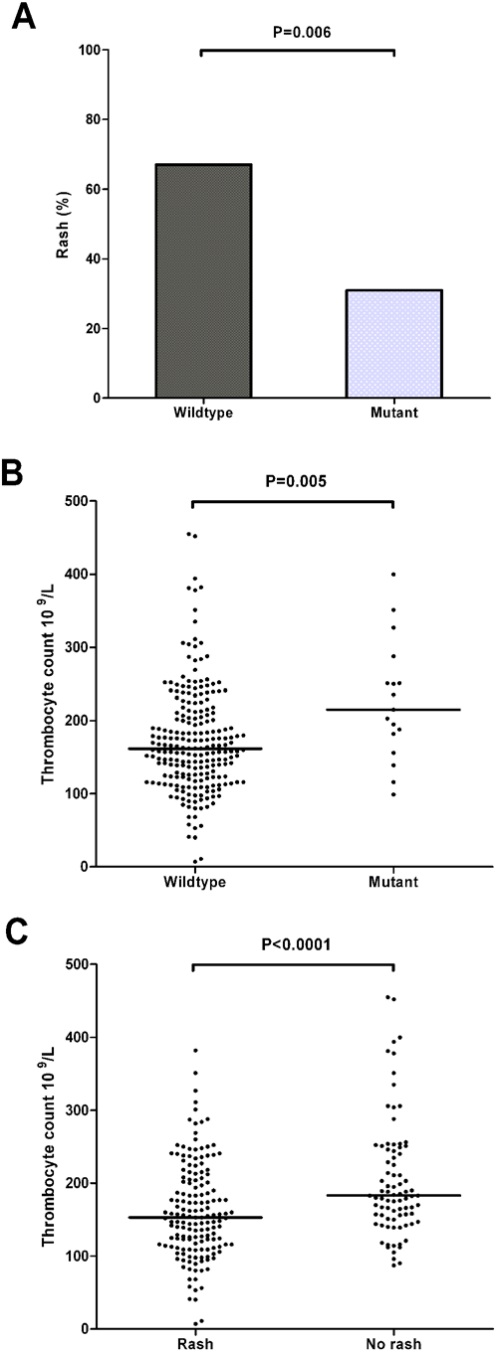
Clinical correlate of *lpxL1* mutations in meningococcal meningitis. (A) Frequency of rash in patients presenting with meningitis infected by *lpxL1* wild-type and mutant strains. (B,C) Platelet counts on admission for *lpxL1* wild-type and mutant strains (B) and patients presenting with and without rash (C). Horizontal bars reflect medians. The Mann-Whitney U test was used to identify differences between groups in continuous variables, and dichotomous variables were compared by the chi-square or Fisher exact test.

Next, we correlated results of the mutation analysis with clinical data (Table 2) [12],[13]. Patients infected with *lpxL1* mutant strains tended to be younger (P = 0.053) and to present less frequently with fever (P = 0.057). None of the patients infected with an *lpxL1* mutant strain presented with hypotension and these patients had correspondingly lower levels of serum creatinine. They were less likely to present with rash compared with those infected with wild-type meningococci (5/16 [31%] vs. 157/236 (67%); P = 0.006; Figure 6) and had higher platelet counts (P = 0.005). Rash was strongly related with lower platelet counts (P<0.0001). To investigate the possibility that the clinical differences found between the two patient groups were confounded by the different ages of the patient groups, a multivariate analysis adjusting for age was performed. The difference in platelet count (P = 0.003) and rash (P = 0.004) remained statistically significant after adjusting for age. Subgroup analysis of clonal complex 41/44 showed similar results. The differences in platelet count (P = 0.007), rash (P = 0.006), and age (P = 0.053) between patients infected by mutant and wild-type strains were also present in the subgroup of clonal complex 41/44.

**Table 2:** Clinical features of 254 adults with meningococcal meningitis due to *lpxL1* mutant and wild-type strains.

Characteristic	Wild-type (N = 237)	*lpxL1* mutant^e^ (N = 17)	P-value^f^
**Admission**			
Age in yr - median (IQR^a^)	31 (19–51)	21 (19–30)	0.053
Fever (temperature >38.0°C) – no. (%)	87/231 (38)	2/16 (13)	0.057
Neck stiffness – no. (%)	205/234 (88)	17/17 (100)	0.23
Median score on GCS^b^ (IQR)	13 (10–15)	12 (11–15)	0.97
Rash	157/236 (67)	5/16 (31)	0.006
Hypotension (Systolic BP^c^ <90 mmHg) – no. (%)	23/204 (11)	0/16 (0)	0.39
Focal neurological deficits – no. (%)	51/237 (22)	2/17 (12)	0.54
**Laboratory investigations**			
Cerebrospinal fluid white cell count – median per mm^3^ (IQR)	5205 (1466–12605)	5376 (3063–11416)	0.89
Positive blood culture – no. (%)	117/207 (49)	11/16 (69)	0.44
Platelet count – median 10^9^/L (IQR)	162 (123–211)	215 (169–270)	0.005
Serum creatinine – median µmol/L (IQR)	95 (77–128)	84 (69–99)	0.051
**Clinical course**			
Septic shock – no. (%)^d^	30/237 (13)	0/17 (0)	0.23
Neurologic complication – no. (%)	98/237 (41)	5/17 (29)	0.45
**Outcome**			
Death – no. (%)	18/237 (8)	1/17 (6)	1.00
Focal neurological deficits – no. (%)	25/218 (11)	3/16 (19)	0.42
Unfavourable outcome – no. (%)	27/237 (11)	3/17 (18)	0.43

a IQR denotes interquartile range.

b GCS Glasgow Coma Scale.

c BP blood pressure.

d Septic shock was defined as systolic blood pressure <90 mmHg with positive blood culture. Systolic blood pressure was measured on admission in 243 patients, GCS in 253 patients, CSF white cell count in 238 patients, and serum platelet count in 241 patients.

e In 16 strains a mutation in *lpxL1* was found, but not in strain 992008.

f The Mann-Whitney U test was used to identify differences between groups in continuous variables, and dichotomous variables were compared by the chi-square or Fisher exact test.

None of the patients infected with *lpxL1* mutant strains developed septic shock during clinical course, while 13% of the wild-type-infected patients did. One patient infected with an *lpxL1* mutant strain died of respiratory failure after multiple seizures. By contrast, sepsis was the leading cause of death among patients infected with wild-type meningococci (14 of 16 fatalities, 88%).

Thus, the *lpxL1* mutation occurs frequently among meningococci causing meningitis. Patients infected by mutant strains have a clinical phenotype consistent with less systemic inflammation and reduced activation of the coagulant system.

## Discussion

Overall, we screened meningococcal isolates of 464 different patients and identified 40 strains with an *lpxL1* mutation. An additional low-activity strain had no *lpxL1* mutation but appeared to be LPS-deficient; the responsible mutation is currently under investigation. Thus, 8.6% of patients were infected with an *lpxL1* mutant, which is surprisingly common. There are several lines of evidence making it very likely that *lpxL1* mutants arise spontaneously in the host instead of introduction of an *lpxL1* mutation into one or several clones and subsequent spreading among the meningococcal population. Firstly, *lpxL1* mutations exist in isolates of many serogroups and clonal complexes. Secondly, we identified 12 unique mutations in *lpxL1*. Thirdly, most *lpxL1* mutations (71%) are due to frameshifts in homopolymeric nucleotide tracts, making phase variation likely. Finally, we found evidence for switching from wild-type to *lpxL1* mutant *in vivo* in patients for which multiple isolates were available.

A picture emerges of *N. meningitidis* modulating its lipid A structure under selective pressure. Under some conditions, hexa-acyl lipid A has to be beneficial to compensate for enhanced recognition by the innate immune system. Lipid A with six acyl chains can protect bacteria from the antibacterial molecules in mucosal secretions, consistent with the observation that many bacteria inhabiting the respiratory tract and gut still produce hexa-acyl LPS [14]. Chronic inflammation of these environments due to LPS stimulation is probably prevented because epithelial cells express low levels of either TLR4, MD-2, or CD14 at the mucosal surface. On the other hand, the submucosal spaces are normally sterile and the defense cells present there, such as macrophages, dendritic cells, and neutrophils, express all the components of the LPS receptor complex and can therefore respond potently after an encounter with a Gram-negative bacterium [14]. Perhaps for this reason most species of Gram-negative bacteria with hexa-acyl lipid A that inhabit the mucosal surfaces rarely become invasive. On the other hand, many Gram-negative pathogens that cause systemic infection do not produce hexa-acyl lipid A. Most of these bacteria have other habitats than the mucosa and enter the body via nonmucosal routes [6]. A good example is the plague bacillus *Yersinia pestis*. At mammalian body temperature *Y. pestis* normally produces tetra-acyl LPS that is poorly recognized by TLR4. Interestingly, a modified strain that produced hexa-acyl LPS at 37°C was no longer virulent in wild-type mice but fully virulent in TLR4-deficient mice, demonstrating the importance of evasion of TLR4 activation for this bacterium [15]. *N. meningitidis* seems to be one of the exceptions to the general rule that Gram-negative bacteria with hexa-acyl lipid A do not cause systemic disease. However, our observation that a proportion of clinical isolates have penta-acylated LPS suggests that evasion of TLR4 activation might aid the bacterium to circumvent host defences after crossing the nasopharyngeal epithelium. The hypothesis that TLR4 plays an important role in the prevention of meningococcal disease corroborates with the finding that subjects with rare TLR4 mutations have an increased risk for developing the disease [16]. If the assumption is correct that hexa-acyl LPS gives the bacterium an advantage on mucosal surfaces and that non hexa-acyl LPS is better for bacteria in submucosal spaces, one would expect that the frequency of *lpxL1* mutants is lower in meningococcal isolates from the respiratory tract compared to meningococcal isolates from the cerebrospinal fluid or blood.

Mogensen et al. showed that strain HF13 is specifically defective in activation of the MyD88-independent pathway, but not in inducing the MyD88-dependent pathway [11]. However, we demonstrate that strain HF13 and other *lpxL1* mutants are also defective in inducing the MyD88-dependent cytokines IL-6, TNF-α, and IL-1β. Our experiments indicate that *lpxL1* mutants or purified *lpxL1* LPS compared to wild-type controls are not specifically deficient in inducing the MyD88-dependent vs. independent pathway. This apparent discrepancy might be explained by the dose of bacteria used. If cells are stimulated with a high dose of bacteria the difference between *lpxL1* mutant and wild-type is only detectable for the MyD88-independent pathway. This is because LPS is the only bacterial component capable of inducing the MyD88-independent pathway, while many other bacterial components can induce the MyD88-dependent pathway (e.g. TLR2 ligands). When cells are stimulated with lower doses of bacteria the difference in induction of the MyD88-dependent pathway becomes apparent, because LPS is by far the most active component of the bacterium and the other non-TLR4 ligands that can activate the MyD88-dependent pathway are diluted too far to be still active.

The relatively high frequency of phase variation raises the question whether the *lpxL1* mutations might have arisen *in vitro* after isolation from the patient. Previously, we have performed extensive research on the phase variation of *porA* in *N. meningitidis*. In this gene, homopolymeric nucleotide tracts are found in the promoter (polyguanidine) and in the coding region (polyadenine). The frequencies by which these sequences vary in length are 10^−3^ [17],[18]. Others showed phase variation of capsule expression caused by insertion of IS1301 in the *siaA* gene with a frequency of phase variation of 9×10^−4^ [19],[20]. *In vitro* selection of *porA* phase variants and *siaA* phase variants have not been reported. Meningococcal isolates received by the Netherlands Reference Laboratory for Bacterial Meningitis (NRLBM) are low passages (up to 2 passages). We sequenced the *lpxL1* gene of 20 individual colonies of a culture of a mutant isolate (971859 I) and of 25 individual colonies of a culture of isolate 971859 III and found in each instance the same sequence, i.e. 20 mutant sequences and 25 wild-type sequences, respectively. Therefore, we estimate the frequency of phase switching to be less than 2.2×10^−2^. In addition, we sequenced *lpxL1* of DNA extracted from a swap taken from 4 different quadrants of another culture plate of isolate 971859 III. All 4 *lpxL1* sequences were homogeneous and identical. Thus we are confident that the discovered *lpxL1* mutations are not caused by *in vitro* phase variation.

Infection with *lpxL1*-mutant meningococcal strains is associated with a particular clinical phenotype, which consisted of less systemic inflammation and reduced activation of the coagulant system, reflected in less fever, higher serum platelet counts, and lower numbers with rash. Moreover, our *in vitro* data have shown that *lpxL1* mutants induce much less pro-inflammatory cytokines than wild-type strains. The coagulation system is activated through upregulation of tissue factor [1]. It has been demonstrated that LPS upregulates tissue factor on monocytes and endothelial cells [21]–[23]. Furthermore, in particular the pro-inflammatory cytokine IL-6 appears to mediate *in vivo* expression of tissue factor [24],[25]. Finally, IL-1β and TNF-α inhibit anticoagulant pathways by downregulating thrombomodulin at the endothelial surface and by increasing plasminogen activator inhibitor type-1 (PAI-1) [26],[27]. Thus, our finding that patients infected with an *lpxL1* mutant show less activation of the coagulation system is consistent with our results that show that *lpxL1* LPS is less potent and that *lpxL1* mutants induce less pro-inflammatory cytokines.

Remarkably, the *lpxL1* mutants induced the same degree of CSF leukocytosis as wild-type strains. There are several explanations for “normal” CSF white cell counts in patients infected by mutant strains. Patients in the cohort all had positive CSF cultures; almost all had clinical signs of meningitis and CSF leukocytosis. Likely, leukocytosis is not only mediated by lipid A, but also by other microbial constituents.

It should be noted that not all groups of patients were included in our analysis of clinical patient data. The study only included adults with meningitis. Patients younger than 16 years or patients with sepsis only were not included. Therefore, our results are potentially biased by excluding these patient groups. Patients with meningitis often have a less severe form of the disease, as reflected by the overall low mortality of 8% in our study. However, patients with sepsis have very serious symptoms resulting from high concentrations of bacteria in the circulation. Mortality rates in these patients can be as high as 50%. Also, patients younger than 16 years are an import group, because rates for meningococcal disease are highest for young children [1]. It would be interesting to see whether *lpxL1* mutants also exist in these patients groups, and if so, if these patients have a different clinical course compared to patients infected with a wild-type strain. These additional data are needed to fully understand the impact of *lpxL1* mutations on meningococcal disease.

Meningococcal sepsis is generally seen as the prototypical endotoxin-mediated disease. Here we report for the first time that meningococcal lipid A mutants which are defective in TLR4 activation occur naturally. Their frequency is unexpectedly high, suggesting an important role in virulence for the resulting low-activity LPS. Our results suggest that in most cases this mutation has occurred through phase variation, and may give the bacteria an advantage because they are less well sensed by the innate immune system. Patients infected with these mutant strains endure milder symptoms with less systemic inflammation and reduced activation of the coagulant system, showing that our findings are clinically relevant. Importantly, these results with *lpxL1* also provide the first example of a specific bacterial mutation which can be associated with the clinical course of meningococcal disease. More generally, it shows how there can be an underestimated heterogeneity in the TLR4-activating capacity of pathogenic bacteria.

## Materials and Methods

### Ethics statement

This observational study with anonymous patient data was carried out in accordance with the Dutch privacy legislation. Written informed consent to use data made anonymous was obtained from the patient (if possible) or from the patient's legal representative.

### *N. meningitidis* strains

Strain HF13 was a kind gift from M. Kilian. The constructed *lpxA* and *lpxL1* mutants were generated in the H44/76 strain as previously described [8],[9]. All other strains were selected from the collection of the Netherlands Reference Laboratory for Bacterial Meningitis. Details about year of isolation, serogroup, genotype and anatomical site of isolation are presented in Table S1. Meningococci were cultured in GC broth or on GC plates (Difco laboratories) supplemented with 1% (vol/vol) Vitox (Oxoid) at 37°C in humified atmosphere of 5% CO_2_ [18]. Bacteria were suspended in PBS and the A_620_ was determined. The bacteria were heat inactivated at 56°C for 30 min. Serogrouping were performed as described elsewhere [12]. MLST was performed as described by Maiden et al [28].

### Lipid A structure

Bacteria were grown as described above and suspended in isobutyric acid-ammonium hydroxide 1 M (5∶3, v/v). Lipid A was extracted as described previously [29] with slight modifications. The lipid A structure was analyzed by nanoelectrospray tandem mass spectrometry (MS/MS) on a Finnigan LCQ in the negative (MS) or positive (MS/MS) ion mode [30].

### Sequencing

DNA was extracted from boiled cultures of *N. meningitidis*. Sequencing of *lpxL1* was carried out using primers 344-2 and 670-1 (Table S2) and BigDyeTerminator chemistry (Applied Biosystems) according to the instructions of the manufacturer. The primers used to obtain sequences upstream and downstream of *lpxL1* are presented in Table S2. Sequence traces were obtained with ABI Big-dyes and an ABI 3730 sequencer.

### Cell lines and PBMCs

PBMC from HLA-oligotyped donors after leukapheresis were isolated by centrifugation of buffy coat cells on Ficoll-Hypaque (Pfizer) and were used after cryopreservation. For experiments and/or maintenance, the human monocyte cell line Mono-mac-6 (MM6), the mouse macrophage cell line J774A.1, and PBMCs were suspended in IMDM (Gibco BRL) supplemented with 100 units/ml penicillin, 100 µg/ml streptomycin, 300 µg/ml l-glutamine (Gibco BRL), and 10% heat-inactivated fetal calf serum (FCS) (Gibco BRL). For experiments and maintenance of HEK-293 cells stably transfected with human TLR4A, MD-2, and CD14 (Invivogen), DMEM (Gibco BRL) was used, supplemented with 10% FCS, 10 µg/ml blasticidin (Invivogen), and 50 µg/ml Hygromycin B (Invivogen).

### ELISA

Depending on the experiment either J774A.1, MM6, PBMCs, or HEK-293 hTLR4/MD-2/CD14 cells were used. Different plates and quantities of cells were used: 1.10^6^ cells in 1 ml medium per well in 12-well plates, 9.10^4^–5.10^5^ cells in 250–1000 µl medium per well in 24-well plates, and 1.10^5^–3.10^5^ cells in 200–300 µl medium per well in 96-well plates. Cells were stimulated with bacteria and incubated o/n at 37°C in a humidified atmosphere containing 5% CO_2_. Cytokine concentrations in the culture supernatants were quantified with ELISA. Mouse IP-10 was determined with mouse IP-10 ELISA kit (R&D systems) and human IL-6, TNF-α, IL-1β, and IL-8 with PeliPairTM reagent sets (Sanquin).

### Meningitis cohort study

The Dutch Meningitis Cohort Study included 258 patients with meningococcal meningitis; from 254 patients the bacterial strain was stored in the Netherlands Reference Laboratory for Bacterial Meningitis [13]. Inclusion and exclusion criteria have been described extensively elsewhere [12]. In summary, eligible patients were older than 16 years, had bacterial meningitis confirmed by culture of cerebrospinal fluid (CSF), and were listed in the database of the Netherlands Reference Laboratory for Bacterial Meningitis from October 1998 to April 2002. This laboratory receives CSF isolates from about 85% of all patients with bacterial meningitis in the Netherlands. The treating physician was contacted, and informed consent was obtained from all participating patients or their legally authorized representatives. This observational study with anonymous patient data was carried out in accordance with the Dutch privacy legislation. Patients underwent a neurologic examination at discharge, and outcome was graded with the Glasgow Outcome Scale. This measurement scale is well validated with scores varying from 1 (indicating death) to 5 (good recovery). A favourable outcome was defined as a score of 5, and an unfavourable outcome as a score of 1–4. Focal neurologic deficits were defined as focal cerebral deficits (aphasia, monoparesis, or hemiparesis) or cranial nerve palsies. Serogrouping, MLST, and susceptibility testing of meningococcal isolates were performed by the Netherlands Reference Laboratory for Bacterial Meningitis.

### Statistics

The Mann-Whitney U test was used to identify differences between groups in continuous variables, and dichotomous variables were compared by the chi-square or Fisher exact test. All statistical tests were 2-tailed, and a p value less than 0.05 was regarded as significant.

### List of accession numbers/ID numbers for genes mentioned in the text

Please see Table S3 for accession numbers.

## Supporting Information

Figure S1 Screening of *N. meningitidis* group Y clinical isolates on cytokine induction. J774A.1 cells were stimulated for 3 h with a panel of group Y strains (0.1 OD) and IP-10 in the supernatant was determined with ELISA.
(2.74 MB TIF)

Figure S2 Screening of panel of *N. meningitidis* clinical isolates representing all major serogroups and clonal complexes. MM6 cells were stimulated for 18 h with a selection of clinical isolates (0.001 OD) representing all serogroups and clonal complexes. IL-6 was determined in the supernatant with ELISA.
(4.22 MB TIF)

Figure S3 Screening of panel of multiple isolates per patient. MM6 cells were stimulated for 18 h with a panel of clinical isolates (0.001 OD), of which multiple isolates were obtained from a single patient. IL-6 was determined with ELISA.
(4.43 MB TIF)

Figure S4 Screening of clinical isolates of patients included in the Dutch meningitis cohort study. MM6 cells were stimulated for 18 h with 254 isolates from patients with meningitis (0.001 OD). IL-6 was determined with ELISA.
(7.22 MB TIF)

Table S1 List of meningococcal strains used in this study.
(0.08 MB XLS)

Table S2 List of primers used for the sequencing of *lpxL1*.
(0.03 MB DOC)

Table S3 List of accession numbers/ID numbers for genes mentioned in the text.
(0.06 MB DOC)
