# Supplementary material for: Naturally Occurring Lipid A Mutants in Neisseria meningitidis from Patients with Invasive Meningococcal Disease Are Associated with Reduced Coagulopathy
Source: PLoS Pathog. 2009 Apr 24;5(4):e1000396. doi: 10.1371/journal.ppat.1000396 (PMC2667671; doi:10.1371/journal.ppat.1000396)
Supplement: Table S2 — List of primers used for the sequencing of lpxL1. (0.03 MB DOC) [file ppat.1000396.s006.doc]

**Table S2. List of primers used for the sequencing of *lpxL1*.**

| **Primer** | **Sequence coordinates**  **(according to MC58 sequence)^a^** |
| --- | --- |
| AvdE_02NMB1417F | 1452953 - 1452971 |
| 344-2 | 1453690 - 1453709 |
| AvdE_NMB1419R | 1454685 - 1454669 |
| 670-1 | 1454693 - 1454674 |
| AVDE-LPX1-101 | 1454977 - 1454960 |
| AVDE_LPX1_100 | 1455059 - 1455039 |

^a^accession number AE002098.2.
