# Supplementary material for: Naturally Occurring Lipid A Mutants in Neisseria meningitidis from Patients with Invasive Meningococcal Disease Are Associated with Reduced Coagulopathy
Source: PLoS Pathog. 2009 Apr 24;5(4):e1000396. doi: 10.1371/journal.ppat.1000396 (PMC2667671; doi:10.1371/journal.ppat.1000396)
Supplement: Table S3 — List of accession numbers/ID numbers for genes mentioned in the text. (0.06 MB DOC) [file ppat.1000396.s007.doc]

**Table S3**

| **Strain Number** | **Accession number** |
| --- | --- |
| 992073 | FJ472279 |
| 9718866 | FJ472280 |
| 9821956 | FJ472281 |
| 2040760 | FJ472282 |
| 2011169 | FJ472283 |
| 2010151 | FJ472284 |
| 2000569 | FJ472285 |
| 2021270 | FJ472286 |
| 2030162 | FJ472287 |
| 2041268 | FJ472288 |
| 2041396 | FJ472289 |
| 2050093 | FJ472290 |
| 2050806 | FJ472291 |
| 2051372 | FJ472292 |
| 2071416 | FJ472293 |
| 2050392 | FJ472294 |
| 970455 | FJ472295 |
| 2050913 | FJ472296 |
| 971523 | FJ472297 |
| 2000311 | FJ472298 |
| 2000607 | FJ472299 |
| 2010640 | FJ472300 |
| 2011764 | FJ472301 |
| 2011833 | FJ472302 |
| 2012202 | FJ472303 |
| 2020434 | FJ472304 |
| 2020622 | FJ472305 |
| 2020799 | FJ472306 |
| 990344 | FJ472307 |
| 990576 | FJ472308 |
| 991093 | FJ472309 |
| 991174 | FJ472310 |
| 991344 | FJ472311 |
| 991382 | FJ472312 |
| 992008 | FJ472313 |
| 971859_I | FJ472314 |
| 971859_III | FJ472315 |
| 970710_I | FJ472316 |
| 970710_III | FJ472317 |
| 941761_I | FJ472318 |
| 941761_III | FJ472319 |
| 2040608_5# | FJ472320 |
| 2040608_3# | FJ472321 |
| 2011334_5# | FJ472322 |
| 2011334_3# | FJ472323 |
